# Chemical components of Fu brick tea and its potential preventive effects on metabolic syndrome

**DOI:** 10.1002/fsn3.3771

**Published:** 2023-11-07

**Authors:** Honghua Li, Wei Dai, Xinjun Zhang, Jie Lu, Fuhang Song, Hua Li

**Affiliations:** ^1^ Key Laboratory of Geriatric Nutrition and Health, Ministry of Education of China School of Light Industry Beijing Technology and Business University Beijing China; ^2^ Key Laboratory of Forest Ecology in Tibet Plateau (Ministry of Education), Institute of Tibet Plateau Ecology Tibet Agriculture & Animal Husbandry University Nyingchi Tibet China

**Keywords:** extracts from Fu brick tea, Fu brick tea, polysaccharides, preventive effects on metabolic syndrome, small molecules

## Abstract

As living standards advance, an escalating emphasis is placed on health, particularly in relation to prevalent chronic metabolic disorders. It is necessary to explore safe and effective functional foods or drugs. Fu brick tea (FBT) is a kind of dark tea fermented by fungi. The extracts are rich in compounds that can effectively relieve metabolic diseases such as hyperglycemia and hyperlipidemia, protect the liver, improve human immunity, enhance antioxidant activity, and regulate intestinal flora. This paper summarizes the biological activities and mechanisms of the extracts, polysaccharides, and small molecular compounds of FBT, which provides a certain theoretical basis for the rational, systematic, comprehensive development and utilization of the FBT resources. It is expected to develop and apply these active substances in health care products and natural medicines and provide more beneficial and diversified FBT products for human beings.

## Graphical abstract

The extracts of Fu brick tea are rich in compounds, which can effectively relieve metabolic diseases. This paper summarizes the biological activities and mechanisms of the extracts, polysaccharides, and small molecular compounds.

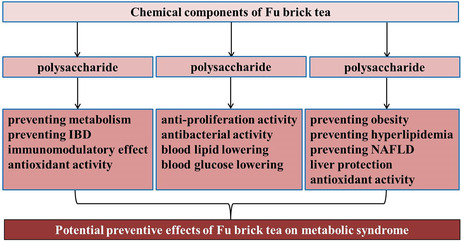

## 1 INTRODUCTION

Fu brick tea (FBT) is a kind of tea produced by microbial fermentation, which is loved by people for its rich and mellow special flavor. FBT is mainly produced in Sichuan, Shaanxi, and Hunan provinces of China. It is the most common drink for ethnic minorities who live in northern and western border areas of China (Chen, Wang, Xie, et al., 2018; Li, Liu, et al., 2013; Tian et al., 2016; Zhang et al., 2017). Under the control of proper temperature and humidity for fermentation, many yellow spores of fungus in tea were notably observed, called “golden flowers” (Li et al., 2021; Li, Lo, et al., 2013; Xiang et al., 2022). During the flowering period, with the growth and reproduction of the dominant strain *Eurotium cristatum*, a series of complex changes have taken place in the biochemical components of FBT, thus forming the unique aroma and taste. Recently, FBT has attracted much attention due to its unique effects of promoting health, such as immune regulation (Sun et al., 2022; Xie et al., 2020), antioxidation (Song & Gao, 2014; Zhao et al., 2022), antiobesity (Li et al., 2022; Li, Liu, et al., 2013; Liu et al., 2015), anti‐diabetes, blood lipid lowering (Li, Lo, et al., 2013; Liu et al., 2020; Liu, Liu, et al., 2022), regulation of intestinal flora (Li et al., 2022; Zhang, Ren, Zhao, Cheng, et al., 2022), liver protection (Foster et al., 2016), antiproliferation (Luo et al., 2013; Tian et al., 2016), and antibacterial (Keller et al., 2013; Wang et al., 2015). FBT has become a common drink in daily life.

Due to the diverse biochemical components and health‐beneficial functions of FBT, the research on FBT has attracted much attention. And many excellent reviews on this subject have been published so far. In 2021, Lin et al. reviewed the microbial fermentation of dark tea, main bioactive compounds, and important biological functions, emphasizing their protective effects against various diseases and relevant molecular mechanisms (Lin et al., 2021). In 2021, Shang et al. reviewed the compounds and health mechanisms of dark teas (Shang et al., 2021). In 2021, Chen et al. reviewed the fermentation processing, microorganisms, chemical constituents, health benefits, and potential risk of FBT (Chen, Bai, et al., 2021). Compared with these reviews, our article focused on FBT, one kind of dark tea, and its diverse compounds. This article reviews some common diseases related to metabolic syndrome (MetS) in recent years (Table 1), including inflammatory bowel disease (IBD), intestinal flora disorder, obesity, hyperlipidemia, nonalcoholic fatty liver disease (NAFLD), diabetes, etc. The urgency of prevention and treatment of these common diseases, the drugs used in clinic, and the existing problems are summarized. These diseases exhibit a high prevalence while suffering from a low therapeutic efficacy. The drugs currently used in clinical have certain limiting effects, but FBT shows potential therapeutic effects on these diseases and has the possibility of developing into prebiotics or drugs (Hu et al., 2018).

**TABLE 1:** FBT shows potential therapeutic effect on some common symptoms.

Symptoms	Urgency of prevention and control	Current drugs for clinical use	Problems in clinical drugs	Therapeutic potential of FBT	References
IBD	The incidence rate has gradually increased in recent years	Azathioprine, aminosalicylic acid, glucocorticoid, etc	There are certain therapeutic limitations, such as substantive side effects	FBT polysaccharide can regulate intestinal flora in patients with IBD	Chen, Chen, et al. (2022), Chen, Wang, et al. (2022), Li et al. (2022)
Alteration of intestinal florar	The imbalance of intestinal flora is closely related to obesity and metabolic diseases such as diabetes and hypertension	Antidiarrheal drugs (montmorillonite powder, somatostatin); microecological preparations (prebiotics, probiotics) etc	Poor specificity, long‐term use of probiotics may lead to dysbacteriosis	FBT can regulate intestinal flora	Zhao et al. (2018)
Obesity	The global incidence rate is increasing year by year, which has been a risk factor for many diseases	Orlistat, sibutramine, metformin, thyroid hormone and its analogues, etc	Olistat is unique, and many drugs are limited due to adverse reactions and safety concerns	FBT has obvious effect on reducing weight and fat	Nudel and Sanchez (2019)
Hyperlipidemia	It is one of the main causes of cardiovascular and cerebrovascular diseases, causing about 17 million deaths worldwide every year, accounting for about 30% of the total deaths	Clofibrate, nicotinic acid, statins, etc	Produce strong dependence, but also produce more side effects after taking the drug. Treatment effect is poor	FBT can regulate transcription factors related to lipid metabolism	Zhou et al. (2022)
NAFLD	The global incidence rate can reach 25.24%	Weight‐reducing drugs, insulin sensitizers, statins, hepatoprotective anti‐inflammatory drugs (vitamin E, ursodeoxycholic acid)	No approved therapeutic drugs	Ingredients from FBT have a certain effect on the prevention and treatment of fatty liver	Chalasani et al. (2018), Younossi et al. (2016)
Diabetes	A common endocrine disease and one of the fastest growing noncommunicable chronic diseases	Sulfonylureas, biguanides, and thiazolidinediones	Single target and pathway, long‐term use causes adverse reactions	FBT and extracts improve insulin resistance symptoms through multiple targets and pathways	Douglas et al. (2020), Singh et al. (2018), Zhu et al. (2021)

In view of the potential activity of FBT on a variety of common diseases, the different chemical components produced by FBT are summarized, including polysaccharides, small molecular compounds, and water/organic solvent extracts (Chen, Chen, et al., 2022). The biological functions and mechanisms of various chemical components are also concluded. This review aims to explore potential safe drugs for relieving and treating obesity, hyperlipidemia, diabetes, and colitis. At the same time, it would be helpful to develop functional food or food additives of FBT to improve human health and prevent diseases.

## 2 BIOLOGICAL ACTIVITY OF FBT POLYSACCHARIDE

In recent years, polysaccharides, as therapeutic agents and mediators of complex cell systems, have become important due to their multiple biological functions. In addition, polysaccharides have little toxic and side effects and have great potential to be developed into medicine or functional food (Chen, Wang, et al., 2022; Chen, Xie, Dai, et al., 2018; Shashidhar et al., 2015). Many studies have shown that complex carbohydrates, which are difficult to digest in the host, are fermented by the flora in the cecum and colon. These carbohydrates regulate intestinal flora by regulating the intestinal flora (Chen, Chen, et al., 2022). Therefore, the digestive process of complex polysaccharides in the intestine is of great significance for regulating immune responses and balancing intestinal flora. FBT polysaccharide is such a typical acid heteropolysaccharide that is difficult to digest (Chen, Li, et al., 2021; Chen, Wang, et al., 2022; Yang et al., 2021). They can directly pass through the upper digestive system and use intestinal flora to reach the large intestine. FBT polysaccharide is the main effective bioactive ingredient, which has biological functions such as weight loss, antioxidation, and immunity enhancement. Therefore, it has been paid more and more attention by people in recent years (Li et al., 2018; Liu, Wang, et al., 2022).

### 2.1 Regulation of FBT polysaccharide on metabolism

FBT polysaccharide can reduce colon tissue damage and inflammation. Researchers found that FBT polysaccharide could reduce ulcerative colitis, reduce the disease activity index of mice, prevent colon shortening, and improve colitis (Chen, Xie, Wan, et al., 2018; Lu et al., 2022; Yang et al., 2021). FBT polysaccharide can regulate the composition of intestinal flora; on the other hand, it can promote the production of shortchain fatty acids as metabolites of intestinal flora, thus improving the intestinal environment. For example, for ulcerative colitis induced by sodium dextran sulfate, FBT polysaccharide can effectively alleviate the intestinal flora disorder caused by colitis, promote the increase of beneficial bacteria such as lactic acid bacteria and Ackermann bacteria, and thus significantly increase the level of short‐chain fatty acids. In addition, it can also improve the tryptophan metabolism of intestinal microorganisms in patients with ulcerative colitis and increase the content of indole‐3‐acetic acid and indole‐3‐acetaldehyde in feces (Yang et al., 2021; Zhang, Ren, Zhao, Shao, et al., 2022).

FBT polysaccharide significantly alleviates MetS in mice induced by a high‐fat diet (Green et al., 2020; Santos‐Marcos et al., 2019). The compositions of intestinal flora are involved in the pathophysiological process of MetS. FBT polysaccharide treatment increased the phylogenetic diversity of intestinal flora in mice induced by a high‐fat diet, and the relative abundance of the Corynebacterium family and Streptococcus family increased. Therefore, the inhibitory effect of FBT polysaccharide on the MetS of mice induced by a high‐fat diet is related to the regulation of intestinal flora. FBT polysaccharide can be used as a candidate drug to prevent MetS related to intestinal flora regulation.

### 2.2 Regulation of FBT polysaccharide on IBD

Polysaccharides can assist in the treatment of IBD (Berg et al., 2013; Chen et al., 2023; Thomas & Morgan, 2013). The anti‐inflammatory activity of natural polysaccharides has gradually become a new way to relieve or treat intestinal inflammation (Xie et al., 2020). FBT polysaccharide can alleviate IBD by regulating intestinal flora disorders, promoting microbial metabolism, and repairing the intestinal barrier.

FBT polysaccharide can improve IBD by regulating intestinal flora and promoting their metabolism. Some researchers studied the relationship between the anti‐inflammatory effect of the purified FBT polysaccharide and intestinal flora (Chen, Chen, et al., 2022; Kang et al., 2019). The intestinal flora of subjects with IBD can utilize and degrade the FBT polysaccharide. At the same time, FBT polysaccharide can regulate the structure of intestinal flora in patients with IBD, making it close to the healthy group. For example, increase the *bacteroides* spp. and reduce the *Escherichia*/*Shigella* (Yang et al., 2021). In addition, the content of short‐chain fatty acids increased significantly. Thus, FBT polysaccharide is expected to be a new probiotic to treat IBD by regulating intestinal flora and promoting the production of short‐chain fatty acids.

FBT polysaccharide can improve IBD by repairing the intestinal barrier. Zeng and Bai et al. studied the anti‐inflammatory activity of crude and purified FBT polysaccharide on mice with colitis induced by sodium dextran (Bai et al., 2022; Zeng et al., 2022). It was found that the expression of lipocalcemin‐2 was significantly reduced in colitis. Both crude and refined FBT polysaccharides could recover the intestinal injury induced by cyclophosphamide. It can restore the intestinal morphology and the expression of tight junction proteins (Occludin, Claudin‐1, and ZO‐1).

### 2.3 Immunomodulatory effect of FBT polysaccharides

The immune system is a reliable defense for protecting human health (Chen et al., 2019; Ding et al., 2019). Some polysaccharides can activate host immune cells and further promote the secretion of a variety of inflammatory cytokines, such as interleukin‐6 (IL‐6), IL‐1β, and tumor necrosis factor TNF‐α, thus exhibiting significant immunomodulatory and anti‐tumor effects (Chen et al., 2023).

Improving immune function is one of the most important biological activities of the FBT polysaccharide. Recently, researchers extracted and purified FBT polysaccharide, which can increase the phagocytosis ability of macrophages RAW264.7, stimulate the secretion of nitric oxide and other inflammatory cytokines (TNF‐α, IL‐1β, IL‐6). It also has obvious immune enhancement activity in vitro. Therefore, FBT polysaccharide can be used as a potential functional food to protect human health by regulating the immune response of the host (Chen, Bai, et al., 2021).

In addition to regulating the immune response by directly acting on immune cells and inflammatory cytokines, FBT polysaccharide can also regulate the immune process by acting on host intestinal microorganisms and secondary metabolites produced by microorganisms (Klausz et al., 2015; Li et al., 2016). FBT polysaccharide has immune protection effect on immunosuppressed mice induced by cyclophosphamide (Bai et al., 2022). It can obviously restore the microbial imbalance caused by cyclophosphamide, increase the abundance of some beneficial bacteria, and reduce the abundance of *Spirobacteriaceae*, *Clostridaceae*, and other strains. The metabonomic analysis showed that FBT polysaccharide significantly changed a series of microbial metabolites, including erucic acid, butyric acid, lysophosphatidic acid, deoxyinosine, taurine, maltotriose, lysophosphatidylcholine, choline, and inosine. These altered metabolites participate in sulfur metabolism, purine metabolism, interaction of neuroactive ligand receptors, phenylpropanoid biosynthesis, protein digestion and absorption, tumor choline metabolism, and glycerol phospholipid metabolism pathways, which are mainly related to antioxidant capacity, immune response, and energy supply of immunosuppressed mice. In addition, there is a significant correlation between specific flora and effective metabolites (Bai et al., 2022). Therefore, FBT polysaccharide can protect the host by regulating intestinal flora and metabolism.

The structural characteristics and immunomodulatory properties of the FBT polysaccharides vary with different extraction methods. Compared with the water extraction of the FBT polysaccharide, the polysaccharide extracted from alkali has a high yield, and the composition of monosaccharide has also changed (Sun et al., 2022). In vitro studies, the FBT polysaccharide extracted from alkali has a significant impact on the acid phosphatase activity, phagocytosis, and nitric oxide secretion of macrophages. In vivo studies, compared with water‐extracted FBT polysaccharide, high doses of alkali extractions showed a considerable or even stronger immune protection and antioxidant activity in the immunosuppressed mice induced by cytoxan. Alkali extracts of FBT polysaccharide can improve intestinal flora composition and intestinal mucosal barrier function, showing a good role in regulating immune imbalance.

### 2.4 Antioxidant activity of FBT polysaccharide

The antioxidant activities of FBT polysaccharide include: the scavenging activity of 1‐diphenyl‐2‐trinitrophenylhydrazine (DPPH) free radical (range: 54.3 ± 1.9~67.8 ± 2.5%), the obvious scavenging activity of superoxide free radical (more than 85%), and the better scavenging activity of ABTS free radical (nearly 100%). In addition, FBT polysaccharide has a protective effect on the oxidative damage of rat pheochromocytoma cells induced by hydrogen peroxide (Chen, Wang, Xie, et al., 2018); FBT polysaccharide can significantly improve the oxidative damage in mice. Therefore, FBT polysaccharide, as a natural and safe antioxidant, has potential application prospects in functional foods.

## 3 SMALL MOLECULE COMPOUNDS AND THEIR ACTIVITIES

In addition to polysaccharides, FBT is also rich in small molecule secondary metabolites, mainly including flavane‐3‐alcohols, noisoprene, acylated flavonoid glycosides, and triterpenes. Some compounds show good biological activity (Chen, Li, et al., 2021; Kong et al., 2019; Lu et al., 2019; Park et al., 2022; Wang, Du, et al., 2021; Zhou et al., 2022).

**FIGURE 1:**
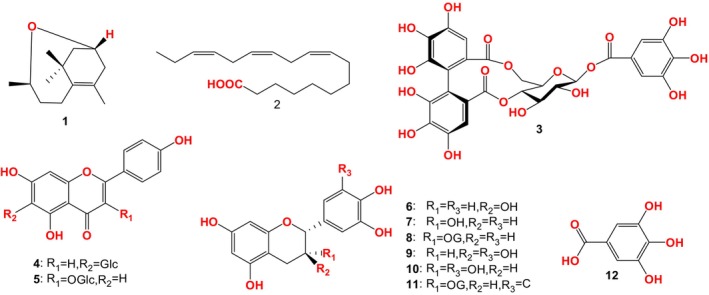
Compounds isolated from FBT by Luo et al. (2012).

12 compounds were isolated from FBT by Luo et al. (Figure 1): 3*R*, 9*R*‐oxido‐5‐megastigmene (1), α‐linolenic acid (2), strictin (3), isovitexin (4), astragalin (5), catechin (6), epicatechin (7), epicatechin gallate (8), gallicatechin (9), galloyl catechin (10), epigallocatechin gallate (11), and gallic acid (12). (Bansal et al., 2013; Luo et al., 2012; Roy et al., 2010; Shi et al., 2021; Tang et al., 2019).

**FIGURE 2:**
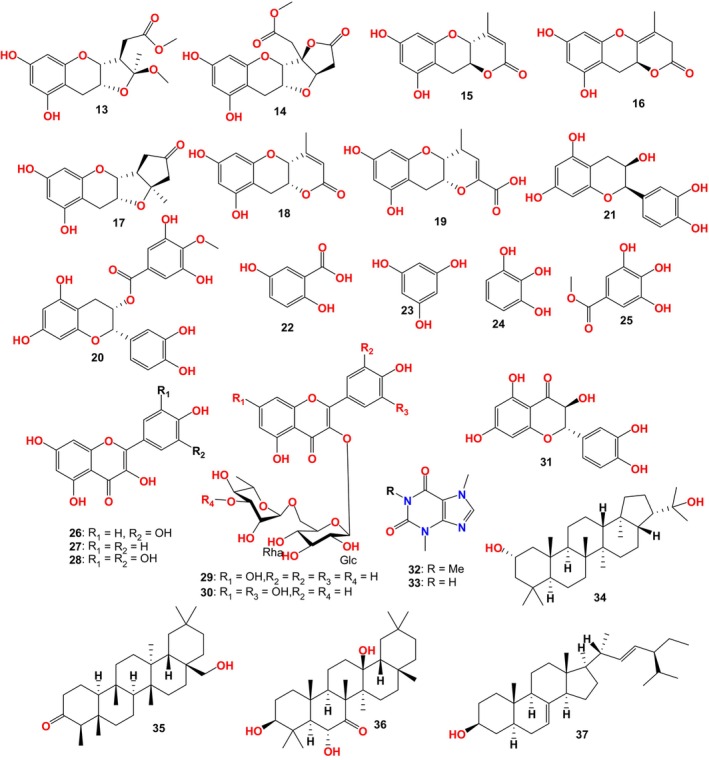
Compounds isolated from FBT by Zhu et al.

31 compounds were separated from FBT (Figure 2), including the B‐ring fission metabolite fuzhuanins C‐F (13–16) of 4 catechins. The B‐ring fission metabolite of 3 planchol A: planchol A (17), xanthocerin (18), teadenol A (19), 6 known catechins: epicatechin (7), epicatechin gallate (8), galloyl catechin (10), epigallocatechin gallate (11), epicatechin‐3‐O‐(4'‐O‐methyl) gallate (20), epiafzelechin (21), 5 mono phenols: gallic acid (12), 2,5‐dihydroxybenzoic acid (22), phloroglucinol (23), pyrogallol (24), gallicin (25), 7 flavonoids and flavonoid glycosides: quercetin (26), kaempferol (27), myricetin (28), astragalin (5), nicotiflorin (29), rutin (30), taxifolin (31), 2 alkaloids: caffeine (32) and theobromine (33), 3 triterpenoids: 2‐hydroxydiplopterol (34), canophyllol (35), 3β,6α,13β‐trihydroxyolean‐7‐one (36) and a steroid α‐spinosterol (37). At present, there is no study on the activity of the compound fuzhuanins C‐F (Boller et al., 2010; Xiao et al., 2022; Zhu et al., 2015).

**FIGURE 3:**
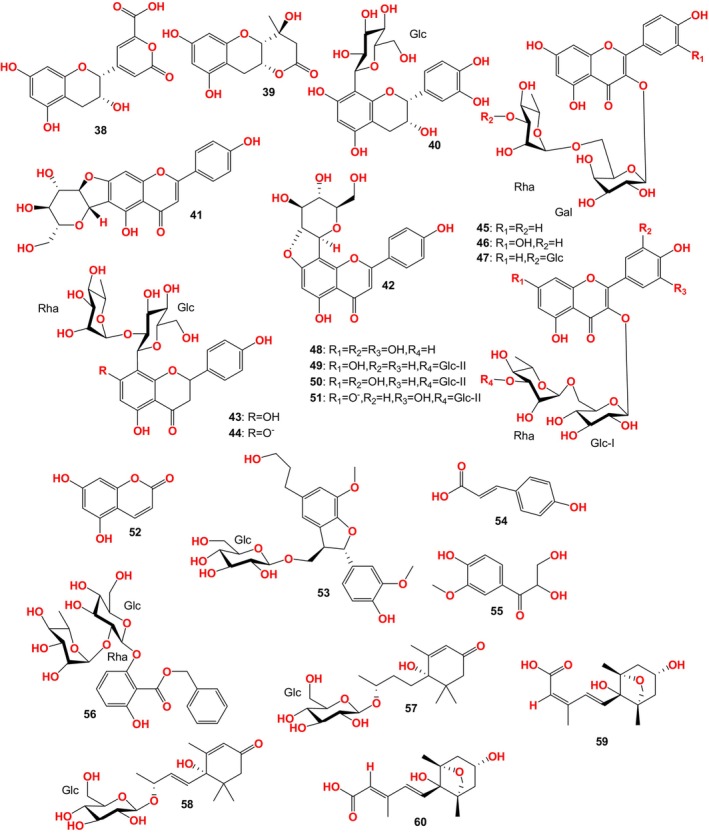
Compounds isolated from FBT by Luo et al. (2013).

28 compounds were separated from FBT by Luo et al. (2013) (Figure 3), including 5 flavane‐3‐alcohols and their derivatives: fuzhuanins A‐B (38, 39), planchol A (17), xanthocerin (18), epicatechin 8‐C‐β‐D‐glucopyranoside (40), 3 flavonoid C‐glycosides: chafurosides A, B (41, 42) and vitexin‐2″‐α‐L‐rhamnopyranoside (43), 8 xanthosides: biorobin (45), quercetin‐3‐O‐robinobioside (46), kaempferol‐3‐O‐[β‐D‐glucopyranosyl‐(1→3)‐O‐α‐Lrhamnopyranosyl‐(1→6)‐O‐β‐D‐galactopyranoside] (47), nicotiflorin (29), rutin (30), myricetin‐3‐O‐rutinoside (48), kaempferol‐3‐O‐[β‐D‐glucopyranosyl‐(1→3)‐O‐α‐Lrhamnopyranosyl‐(1→6)‐O‐β‐D‐glucopyranoside] (49), quercetin‐3‐O‐ [β‐D‐glucopyranosyl‐(1→3)‐O‐α‐L‐rhamnopyranosyl‐(1→6)‐O‐β‐D‐glucopyranoside] (50), 5 mono phenols: 5,7‐dihydroxycoumarin (52), (7*R*, 8*S*)‐dihydrodeoxiconiferol alcohol 9‐O‐β‐D‐glucopyranoside (53), p‐coumaric acid (54), 2,3‐dihydroxy‐1‐ (4‐hydroxy‐3‐methoxyphenyl)‐propan‐1‐one (55), benzyl‐2‐neohesperidosyloxy‐6‐ hydroxybenzoate (56), 2 isoprene reducing glycosides: roseoside (57) and icariside B_5_ (58), 2 sesquiterpenoids: dihydrophaseic acid (59), 5‐(3,8‐dihydroxy‐1,5‐dimethyl‐6‐oxabicyclo[3.2.1]oct‐8‐yl)‐3‐methyl‐2(*E*),4(*E*)‐pentadienoic acid (60), 1 theobromine (33), and 2 flavonoid anions: vitexin‐2″‐α‐ L‐rhamnopyranosyl‐7 (44) and quercetin‐3‐O‐[β‐D‐glucopyranosyl‐(1→3)‐O‐α‐L‐rhamnopyranosyl‐(1→6)‐O‐β‐D‐glucopyranoside] ‐7‐oxygen anion (51). Fuzhuanin A was one of the main characteristic components produced during fungal fermentation. The IC_50_ value of fuzhuanin B anti proliferation activity was 4.48 μM in HeLa cells (Boller et al., 2010; Li, Liu, et al., 2013; Luo et al., 2013; Mahdavi‐Roshan et al., 2020).

**FIGURE 4:**
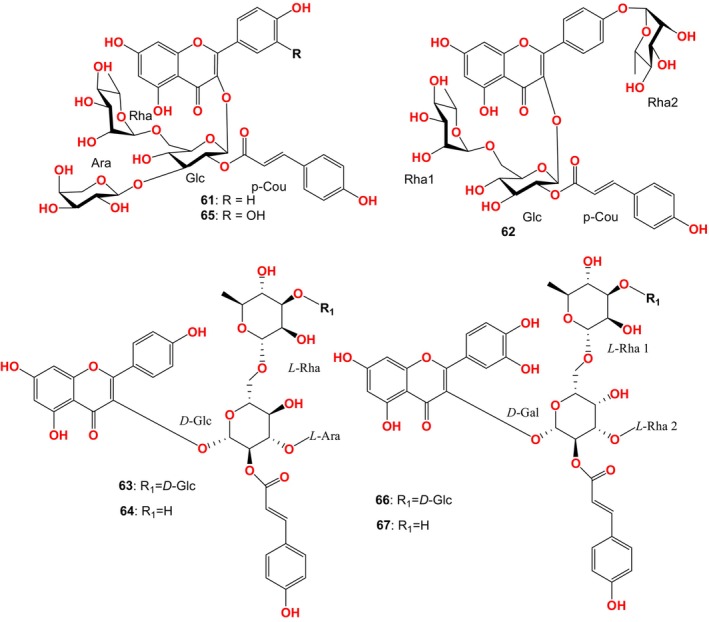
Compounds isolated from FBT by Tian and Lu et al.

Sven new acylated flavonoid glycosides (Figure 4) were separated from FBT, including 4 quercetyl glycosides and 3 kaempferide glycosides: camellikaempferosides A, B, D, E (61–64) and camelliquercetisides C, E, F (65–67). Flavonoid glycosides and their metabolites participated in the regulation of hypoglycemic and hypolipidemic effects. Camellikaempferoside A had antiproliferation activity against MDA MB‐231 and MCF‐7 cells with IC_50_ values of 19.16 and 7.83 μM, respectively (Tian et al., 2016). Camellikaempferoside B could inhibit the formation and aggregation of β‐amyloid protein. It also improved the β‐amyloid‐induced neuronal cell death, production of reactive oxygen species, release of inflammatory factors, and activation of microglia. It had the potential application value in the development of therapeutic drugs for Alzheimer's disease (Xu, Hu, et al., 2019; Yang et al., 2016). Camelliquercetisides E, F and camellikaempferosides D, E had good α‐glucosidase and HMG CoA reductase inhibitory activity (Bai et al., 2017; Lu et al., 2019).

**FIGURE 5:**
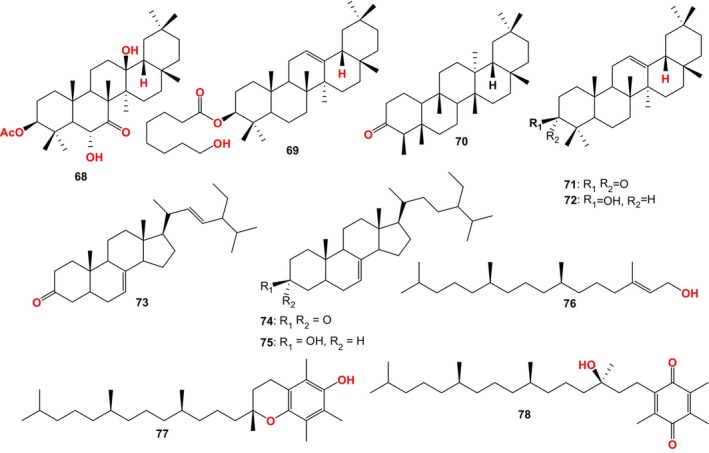
Compounds isolated from FBT by Ling et al.

Fourteen compounds were isolated from FBT by Ling et al. (Figure 5), including 3 new triterpenoids: 3β,6α,13β‐trihydroxyolean‐7‐one (36), 3β‐acetoxy‐6α,13β‐dihydroxyolean‐7‐one (68), and 3β‐O‐(8‐hydroxyoctanoyl)‐12‐oleanene (69), 11 known compounds: friedelin (70), β‐amyrone (71), β‐amyrin (72), α‐spinasterone (73), α‐spinosterol (37), 22,23‐dihydrogen‐α‐boresterone (74), 22,23‐dihydro‐α‐spinasterol (75), α‐phytol (76), α‐tocopherol (77), α‐tocoquinone (78), caffeine (32). Among them, 3β,6α,13β‐trihydroxyolean‐7‐one had antibacterial activity against some intestinal pathogenic microorganisms (Ling et al., 2010). The MIC values against enteropathogenic *Streptococcus typhi* and *Escherichia coli* were 800 and 400 μg/mL, respectively. The inhibitory activity against *dysentery bacillus* was equivalent to that of berberine hydrochloride, with a MIC value of 100 μg/mL (Keller & Wallace, 2021; Kondo et al., 2018; Ling et al., 2010).The compounds may be a potentially safe anti‐dysentery drug or lead compound.

To sum up, abundant small‐molecule compounds were isolated from FBT. Some of the compounds showed biological activity, among which flavonoids, glycosides and their metabolites were involved in regulating hypoglycemia and lowering blood lipids. In addition, fuzhuanin B (39) and camellikaempferoside A (61) had antiproliferation activity. 3β,6α,13β‐trihydroxyolean‐7‐one (36) had antibacterial activity against some intestinal pathogenic microorganisms. The results of the current study show that the role of small molecular compounds in regulating metabolic diseases is weak.

## 4 BIOLOGICAL ACTIVITY OF FBT EXTRACTS

Nowadays, FBT has become an indispensable drink in northwest China, and there is a saying that “it is better to be without food for three days than without tea for one day” (Emami Arjomand et al., 2021; Hayat et al., 2015; Xu et al., 2022; Zheng et al., 2022). In order to further analyze its functional components, the researchers obtained the organic solvent and water extracts of FBT and found that the extracts had various components, including polyphenols, amino acids, organic acids, polysaccharides, and pigments. In addition to forming the unique flavor and aroma of FBT, it also made FBT have a variety of significant biological activities, such as weight loss, blood lipid reduction, anti‐oxidation, and antibacterial (Li, Lo, et al., 2013; Liu et al., 2015).

### 4.1 Regulatory effect of FBT extracts on obesity and hyperlipidemia

In the past decades, obesity has become an urgent global public health problem, contributing to heart disease, hyperlipidemia, hypertension, diabetes, and fatty liver (Zhou et al., 2022; Zhou, Li, et al., 2021). FBT extracts have the potential function of preventing obesity. For example, the water extracts of FBT can significantly inhibit the fat deposition of adipocytes in *Caenorhabditis elegans* (Peng et al., 2014). In addition, the water extracts of FBT can also significantly inhibit the weight increase and adipose tissue accumulation of obese rats induced by a high‐fat diet. And the water extracts could also reduce the levels of total cholesterol, serum triacylglycerol, and low‐density lipoprotein. Thus, FBT extracts have significant effects on weight loss and blood lipid reduction (Li, Liu, et al., 2013; Zeng et al., 2022; Zhou, Tian, et al., 2021).

The mechanism of weight loss and blood lipid reduction of FBT extracts is the result of the joint action of different genes (Goldschmidt et al., 2011; Li et al., 2019; Liu et al., 2019; Wilfley et al., 2017): the energy consumption and lipid conversion can be increased by improving the expression of fatty acid oxidation‐related genes, such as liver peroxisome proliferator‐activated receptor α and carnitine palmitoyltransferase 1α; lipid storage in tissues can be reduced by enhancing the expression of cholesterol clearance‐related genes in the blood, such as low‐density lipoprotein receptor; fat synthesis can been reduced by inhibiting the expression of lipid metabolism‐related genes, such as fatty acid synthase, sterol regulatory element binding protein‐1C, and CCAAT/enhancer binding protein α (Li, Lo, et al., 2013).

### 4.2 Regulatory effect of FBT extracts on NAFLD

In recent years, FBT has shown a curative effect on NAFLD and related MetS. The water extracts of FBT can reduce the effect of high‐fat diet on the rat liver and kidney (Du et al., 2019; Li, Liu, et al., 2013). Fat production of rats fed with FBT extracts was reduced. Meanwhile, β‐oxidation tricarboxylic acid circulation, and respiratory chain increased, which mainly contributed to improving NAFLD‐related liver fat accumulation. A large amount of protein is involved in the metabolism of sugar and lipids, such as fatty acid synthase and other proteins mentioned above.

Further research shows that the water extracts of FBT have free radical scavenging activity, good glycosidase inhibition, alleviation of insulin resistance, and hypoglycemic activity in HepG2 cells in type 2 diabetes mice and can activate the receptor cascade signal pathway activated by phosphatidylinositol 3‐kinase‐Akt‐peroxisome proliferation. Regulate glycolipid metabolism and change the activities of key enzymes related to glycolipid metabolism (Zhu et al., 2021).

### 4.3 Protective effect of FBT extracts on liver

The water extracts of FBT can reduce the liver dysfunction and intestinal flora imbalance of the high‐fructose fed mice (Wang et al., 2015; Zhang et al., 2019). It has a strong inhibitory effect on dyslipidemia, liver weight increase, serum enzyme activity, and the formation of liver inflammatory cytokines in mice fed with high fructose. The treatment of water extracts of FBT reduced the formation of malondialdehyde in the liver and increased the activities of glutathione peroxidase and superoxide dismutase. At the same time, the abundance of *Bacteroidetes* in the intestinal tract of high‐fructose fed mice was significantly increased, and the number of *Firmicutes*, *Proteobacteria* was decreased (Chen, Xie, Dai, et al., 2018; Zhang et al., 2019). These results indicate that the water extracts of FBT can protect the liver by improving oxidative stress, inflammatory reaction, and intestinal flora dysfunction (Chai & Jung, 2022).

### 4.4 Antioxidant activity of FBT extracts

FBT extracts contain a large number of polysaccharides, polyphenols, and flavonoids, which have good antioxidant activity (Song & Gao, 2014; Zhang et al., 2017; Zhao et al., 2022). In addition, gallic acid, gallate ester, gallocatechin, and epicatechin are also strong antioxidant candidates. The methanol extracts of FBT have a protective effect on the oxidative stress of human intestinal epithelial adenocarcinoma cell Caco‐2 induced by hydrogen peroxide. The methanol extracts can inhibit lipid peroxidation, increase glutathione levels, and antioxidant enzyme activity (Song & Gao, 2014). The n‐hexane extracts of FBT mainly include gallic acid, theaflavin, theobromine, caffeine, epicatechin, and quercetin. They show antiaging effect in cell melanin A and mice and can significantly reduce the production of reactive oxygen species in melanin A cell (Zhao et al., 2022).

Apart from methanol and n‐hexane organic solvent extracts, the water extracts of FBT also show strong antioxidant activity (Xu, Sun, et al., 2019; Zhao et al., 2018). They have antagonistic effects on photoaging of human keratinocytes HaCaT when exposed to ultraviolet B. It can inactivate the production of reactive oxygen species in cells induced by ultraviolet B without any cytotoxicity. The water extracts of FBT can protect the photoaging induced by ultraviolet in HaCaT cells. It can regulate nuclear factor‐related factor 2 and downregulate the expression of matrix metalloproteinase‐1. Therefore, the water extracts of FBT are good candidate ingredients for cosmetics and drugs, which can be used to repair the skin photoaging caused by ultraviolet B.

## 5 CONCLUSION

FBT is a unique post‐fermentation tea product. Fungal fermentation has a significant impact on the biochemical characteristics of FBT (Li et al., 2017; Xia et al., 2022; Xu et al., 2015, 2020). After fermentation, various metabolic products, including polysaccharides and polyphenols, provide a variety of options for the development of functional products of FBT (Darwish & Xie, 2012; Dridi et al., 2010; Patil et al., 2015; Powell‐Cope et al., 2014; Wang, Zhao, et al., 2021). FBT polysaccharide has high antioxidant activity in vitro and maintains human health by regulating metabolic processes and immune activity. In addition, FBT polysaccharide has a regulatory effect on IBD. Therefore, FBT polysaccharide is expected to be a natural antioxidant, providing potential safe drugs for the relief and treatment of colitis. At present, a variety of small‐molecule compounds have been isolated from FBT. Some of them have diverse activities, such as antiproliferation activity, antibacterial activity, blood lipid lowering, and blood glucose lowering effects on Hela and other cells. These compounds mainly included 1 isoprene‐reducing compound, 6 flavan‐3‐alcohols, fuzhuanins A‐F, 7 acylated flavonoid glycosides, and 3 triterpenoids. There are still many unknown compounds that need to be further isolated, purified, and identified. Their beneficial effects on health and underlying mechanisms need to be studied. The water/methanol/n‐hexane extracts of FBT have antioxidant activity. These extracts can significantly regulate obesity, hyperlipidemia, and NAFLD and have a protective effect on the liver. Therefore, FBT extracts have the possibility of developing into prebiotics or drugs. The molecular function mechanisms of FBT are diverse, with MAPK and TNF signaling pathways being the key ones. Some studies have shown that FBT prevents obesity and hyperlipidemia by regulating gut flora. But there is no direct evidence to confirm that.

To sum up, in terms of function, FBT shows rich biological activities (Leonardo et al., 2020; Roth et al., 2022; Sasa et al., 2022; Schur et al., 2020), such as antioxidation, anti‐inflammatory, antimetabolic syndrome, antitumor, regulation of glucose metabolism, regulation of intestinal microorganisms, and improvement of immune activity. However, there are few reports on the isolation and identification of small molecular compounds that can alleviate and treat metabolic diseases. The functional evaluation has been carried out on the small molecular compounds. It can provide a strong guarantee and technical support for the further processing and quality control of FBT products. And there is a need for clinical studies on this subject.

## AUTHOR CONTRIBUTIONS

**Honghua Li:** Funding acquisition (supporting); writing – original draft (lead); writing – review and editing (lead). **Wei Dai:** Writing – review and editing (supporting). **Xinjun Zhang:** Writing – review and editing (supporting). **Jie Lu:** Writing – review and editing (supporting). **Fuhang Song:** Funding acquisition (lead); writing – original draft (supporting); writing – review and editing (supporting). **Hua Li:** Writing – review and editing (supporting).

## FUNDING INFORMATION

This study was supported by the Science and Technology Program of Tibet Autonomous Region [grant number: XZ202101YD0013C], the National Natural Science Foundation of China [grant number: 31960013, 31901741], the General Projects of Science and Technology Program of Beijing Municipal Education Commission [grant number: KM202210011008], Research Foundation for Young Teachers of Beijing Technology and Business University [grant number: QNJJ2022‐21], and the State Key Laboratory of Silkworm Genome Biology.

## CONFLICT OF INTEREST STATEMENT

The authors declare no conflict of interest.

## Data Availability

This review did not report any data.
